# Metabolomics analysis reveals altered metabolites in lean compared with obese adolescents and additional metabolic shifts associated with hyperinsulinaemia and insulin resistance in obese adolescents: a cross-sectional study

**DOI:** 10.1007/s11306-020-01759-y

**Published:** 2021-01-12

**Authors:** Elisabeth Müllner, Hanna E. Röhnisch, Claudia von Brömssen, Ali A. Moazzami

**Affiliations:** 1 Department of Molecular Sciences, Swedish University of Agricultural Sciences, Uppsala, Sweden; 2 Department of Energy and Technology, Unit of Applied Statistics and Mathematics, Swedish University of Agricultural Sciences, Uppsala, Sweden

**Keywords:** NMR metabolomics, Hyperinsulinaemia, Insulin resistance, Obesity, Energy metabolism

## Abstract

**Introduction:**

Hyperinsulinaemia and insulin resistance (IR) are strongly associated with obesity and are forerunners of type 2 diabetes. Little is known about metabolic alterations separately associated with obesity, hyperinsulinaemia/IR and impaired glucose tolerance (IGT) in adolescents.

**Objectives:**

To identify metabolic alterations associated with obesity, hyperinsulinaemia/IR and hyperinsulinaemia/IR combined with IGT in obese adolescents.

**Methods:**

81 adolescents were stratified into four groups based on body mass index (lean vs. obese), insulin responses (normal insulin (NI) vs. high insulin (HI)) and glucose responses (normal glucose tolerance (NGT) vs. IGT) after an oral glucose tolerance test (OGTT). The groups comprised: (1) healthy lean with NI and NGT, (2) obese with NI and NGT, (3) obese with HI and NGT, and (4) obese with HI and IGT. Targeted nuclear magnetic resonance-based metabolomics analysis was performed on fasting and seven post-OGTT plasma samples, followed by univariate and multivariate statistical analyses.

**Results:**

Two groups of metabolites were identified: (1) Metabolites associated with insulin response level: adolescents with HI (groups 3–4) had higher concentrations of branched-chain amino acids and tyrosine, and lower concentrations of serine, glycine, myo-inositol and dimethylsulfone, than adolescents with NI (groups 1–2). (2) Metabolites associated with obesity status: obese adolescents (groups 2–4) had higher concentrations of acetylcarnitine, alanine, pyruvate and glutamate, and lower concentrations of acetate, than lean adolescents (group 1).

**Conclusions:**

Obesity is associated with shifts in fat and energy metabolism. Hyperinsulinaemia/IR in obese adolescents is also associated with increased branched-chain and aromatic amino acids.

**Supplementary Information:**

The online version contains supplementary material available at 10.1007/s11306-020-01759-y.

## Introduction

Worldwide, approximately 23% of children and adolescents in developed countries are overweight or obese and, despite major policy efforts, there are no signs of improvements (Ng et al. 2014). Obese adolescents are at higher risk of developing associated complications such as insulin resistance (IR) (loss of sensitivity to insulin in the peripheral tissues). IR and insulin secretion are coupled with a feed-back mechanism which governs their relationship (Arslanian 2005; Levy-Marchal et al. 2010; Van Name and Caprio 2013). Individuals with IR compensate with higher insulin secretion if they have sufficient beta cell function. However, if beta cell function is impaired/depressed (compensatory increase in insulin secretion is incomplete) in IR individuals, impaired glucose tolerance (IGT) occurs, putting individuals at risk of developing type 2 diabetes (T2DM) (Arslanian 2005; Levy-Marchal et al. 2010). The number of children developing obesity-associated complications (IR, IGT and T2DM) is increasing (Amed et al. 2010) and healthcare costs related to overweight/obesity and its comorbidities are escalating (Lehnert et al. 2013). To address this threat, there is an urgent need for research to identify pathophysiological disturbances associated with different stages on the pathway to T2DM.

Several studies have assessed metabolic differences between lean and obese children in the fasting state (Butte et al. 2015; Farook et al. 2015; Perng et al. 2014; Wahl et al. 2012). Increased plasma levels of branched-chain amino acids (BCAAs), aromatic amino acids (AAAs) and acylcarnitines are repeatedly reported in obese children (for review, see Zhao et al. 2016), although the findings on disturbances in amino acid metabolism are conflicting (Farook et al. 2015; Michaliszyn et al. 2012; Mihalik et al. 2012; Wahl et al. 2012). Studies in which obese individuals are stratified based on IR, e.g. using the homeostasis model assessment of insulin resistance (HOMA-IR), are limited (Mastrangelo et al. 2016). There is accumulating evidence of an obese phenotype (also referred to as ‘healthy’ obese) free of metabolic disorders (e.g. IR, T2DM or abnormal blood lipid profile) (Blüher and Schwarz 2014; Chen et al. 2015). Therefore, it is of great interest to stratify individuals through consideration of both body weight and IR. Such an experimental setup can distinguish the metabolic shifts associated with obesity from those additionally associated with hyperinsulinaemia/IR and provide a better understanding of the metabolic perturbation associated with obesity, IR and T2DM.

Assessment of IR using only fasting samples can have limitations, and inclusion of post-ingestion/challenge samples, i.e. post-oral glucose tolerance test (OGTT), is considered to provide a more accurate estimate of whole-body IR (Borai et al. 2007). Increases in insulin responses with concomitant increases in IR have also been shown in adolescents (Kelsey et al. 2020; Kim et al. 2020) and changes in insulin AUC after an OGTT have been used to monitor changes in IR in response to different treatments in children (Davis et al. 2012). In addition, it has been shown that a metabolic challenge (such as OGTT or meal intake) can increase inter-individual variations and aid in unmasking metabolic phenotypes (Krug et al. 2012). We have previously observed that the magnitude of insulin response to a standard meal is strongly associated with a metabolic signature associated with increased risk of T2DM (Moazzami et al. 2014; Shrestha et al. 2017). Therefore, using post-OGTT samples can be a useful tool for characterising individuals and revealing their metabolic status.

Little is known about the metabolic shifts associated with different body mass index (BMI) values and, among obese adolescents, the metabolic shifts associated with the different stages (i.e. hyperinsulinaemia/IR, IGT) toward T2DM development. Therefore, in the present study, well-characterised adolescents from the Uppsala Longitudinal Study of Childhood Obesity Cohort (ULSCO) (Forslund et al. 2014) were stratified, based on body mass index (BMI), insulin (post-OGTT) and glucose (post-OGTT) concentrations, into four groups in a cross-sectional study (the term “Longitudinal” refers to the name of cohort and is not applicable for the experimental design of the present study). These groups were: (1) healthy lean, (2) obese with normal insulin (NI), (3) obese with high insulin (HI), and (4) obese with HI and impaired glucose tolerance (IGT). Surrogates for insulin resistance (i.e. HOMA-IR and Matsuda Index) were also calculated, using glucose and insulin concentrations before and after OGTT. This allowed metabolic alterations associated with obesity to be distinguished from additional alterations associated with hyperinsulinaemia/IR and hyperinsulinaemia/IR combined with IGT.

## Experimental

### Uppsala longitudinal study of childhood obesity cohort

The ULSCO cohort is an observational cohort study (Forslund et al. 2014) initiated in 2010 that includes obese (BMI ≥ 95th percentile for age and sex) and lean control (BMI < 85th percentile for age and sex) children (≤ 18 years old) according to age and sex-dependent World Health Organization (WHO) growth curves. The children underwent a 2-h OGTT after overnight fasting (1.75 g glucose/kg body weight, maximum 75 g) and blood samples were collected at 0 (fasting), 5, 10, 15, 30, 60, 90 and 120 min. All blood samples were immediately placed on ice and centrifuged at 4 °C for 10 min. Plasma was stored at − 80 °C in a biobank until analysis. The study was performed in accordance with the Helsinki Declaration and approved by Uppsala Regional Ethics Committee (registration numbers 2010/036 and 2012/318). Legal guardians and children ≥ 12 years of age provided informed and written consent. Details of the study protocol have been published previously (Forslund et al. 2014).

### Selection of samples for metabolomics analysis

**Fig. 1:**
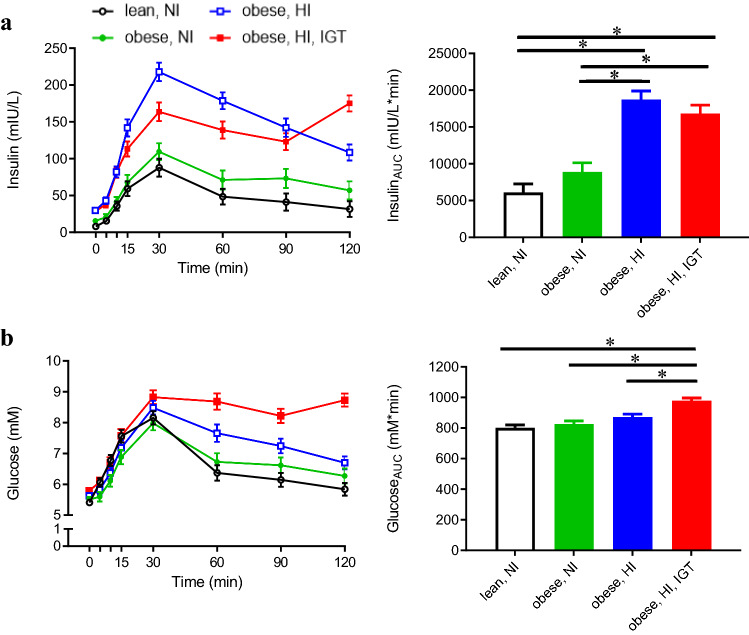
Plasma concentrations during an oral glucose tolerance test (OGTT; left panel) and area under the curve (AUC; right panel) of **a** insulin and **b** glucose in lean adolescents with normal insulin (NI; black, n = 21), adolescents with obesity and NI (green, n = 18), adolescents with obesity and high insulin (HI; blue, n = 20), and adolescents with obesity and HI in combination with impaired glucose tolerance (IGT; red, n = 23) (Color figure online)

For the present metabolomics study, a subgroup of obese and lean adolescents aged ≥ 10 years was selected. Exclusion criteria were intake of medication, syndromic obesity, no OGTT, impaired fasting glucose (IFG; fasting glucose between 6.1 and 6.9 mmol/L (110–125 mg/dL) based on WHO criteria (Alberti and Zimmet 1998), diagnosed diabetes, fasting plasma glucose level ≥ 7 mmol/L (126 mg/dL), and/or 2-h post-OGTT glucose ≥ 11.1 mmol/L (200 mg/dL, WHO criteria) (Alberti and Zimmet 1998). A flow chart showing the number of adolescents excluded due to the above-mentioned reasons, or because their insulin response curves did not meet the criteria defined below, is presented in Online Resource 1. The adolescents were stratified into four groups, based on their BMI, their insulin secretory pattern (time and number of insulin peaks and AUC of insulin) in response to the OGTT, and their 2-h glucose level. Stratification of the adolescents based on these criteria was expected to help in identifying changes in the metabolic profile in different stages on the pathway toward T2DM i.e. obesity, hyperinsulinaemia/IR and IGT. The characteristics of the four groups are shown in Fig. 1 and defined as follows: Group 1: healthy, lean adolescents with BMI < 85th percentile for age and sex, normal glucose tolerance (NGT) and normal insulin secretory response during the OGTT (NI) (Ferrannini 2010), with an insulin peak within the first 30 min and insulin at 60, 90 and 120 min lower than at the peak. Group 2: obese adolescents with NGT and similar insulin secretory response during the OGTT (Ferrannini 2010) as group 1. Group 3: obese adolescents with NGT and a high insulin secretory response during the OGTT (HI) (compared with groups 1–2), the peak of insulin at 30 or 60 min, and insulin at 90 and 120 min lower than at the peak. Group 4: obese adolescents with IGT and high and biphasic insulin secretory response during the OGTT (HI), the first peak of insulin at 30 or 60 min, and the second peak at 90 or 120 min.

### Anthropometry clinical assessments and metabolomics analyses

Details of anthropometric measurements, clinical assessments (i.e. plasma glucose and insulin analyses, calculation of surrogate of insulin resistance (HOMA-IR and Matsuda Index)), data and sample retrieval from ULSCO (Forslund et al. 2014) and targeted quantitative NMR-based metabolomics analyses are available in Online Resource 2.

### Statistical analysis

#### Univariate data analysis

Univariate analyses were performed using Statistical Analysis System software (SAS 9.3, SAS Institute, Cary, NC, USA). Basic characteristics (e.g. age, BMI and fasting insulin) of the four groups were compared by analysis of variance (ANOVA) with Bonferroni *post hoc* test.

Differences in metabolic profile between the groups at the different time points were assessed in five steps. First, a linear mixed model (PROC MIXED) with time, group (1–4) and their interaction (time × group) as fixed factors was run. To adjust for the repeated measures structure of the data, correlations between the observations made on the same individual at different time points were estimated using an unstructured variance-covariance matrix. Normal distribution of residuals was tested, and if data were skewed log transformation was applied. Second, the Benjamini-Hochberg (BH) method (Benjamini and Hochberg 1995) was used to adjust *P*-values (for group and time × group interaction) for multiple testing. *P*-values smaller than the BH-corrected significance level were considered significant. Third, metabolites with significant *P*-values for group and/or time × group interaction after BH correction were further analysed by Bonferroni post-hoc test, in order to identify groups that were significantly different at each single time point. Fourth, gender and pubertal stage were included in the mixed model used in step 1, in order to test whether group and/or time × group effects remained significant after adjustment for gender and puberty. In addition to gender and puberty, the model was further adjusted for BMI for the metabolites found to be significant after BH correction. Fifth, all metabolites which were found to be discriminative after step 2 or multivariate statistical analysis (*vide infra*) (total number of metabolites n = 16) were further investigated by a linear mixed model (PROC MIXED) with time, insulin response (NI vs. HI), obesity (lean vs. obese), glucose response (NGT vs. IGT) and their interactions (time × insulin, time × obesity and time × glucose) as fixed factors and adjusted for gender and puberty. This fifth step was performed to further distinguish between insulin response, obesity, and glucose response effects.

#### Multivariate data analysis

SIMCA 14 software (Umetrics, Umeå, Sweden) was used for multivariate data analysis by principal component analysis (PCA) and partial least squares discriminant analysis (PLS-DA). Unit variance scaling was used considering the quantitative nature of the targeted analysis (as metabolites with both high and low concentration could be of importance in association with different groups). PCA models were used to identify outliers. PLS-DA was used to determine discriminative metabolites between the groups at each time point. The validity and reliability of the PLS-DA models were tested by cross-validated ANOVA (Eriksson et al. 2008), which assesses whether the model has significantly smaller cross-validatory predictive residuals than just the variation around the global average (Eriksson et al. 2008). Models with CV-ANOVA *P*-values < 0.05 were considered significant. Metabolites with (1) variable influence on projection (VIP) values > 1, and (2) VIP jackknife-based 95% confidence intervals (CIs) not close to or including zero were considered discriminative. Metabolites that met these criteria were further evaluated using the first, third, fourth and fifth steps in univariate statistical analysis described in Sect. 2.4.1, with a threshold of *P* < 0.05.

## Results

### Study population

General characteristics of adolescents from the ULSCO cohort selected for metabolomics analyses are shown in Table 1. Mean BMI-standard deviation score (SDS) differed between lean and obese adolescents (obese NI; obese HI; obese HI + IGT). As intended by the study design, fasting glucose and insulin, and glucose and insulin area under the curve (AUC), were not different between lean and obese adolescents with NI (Table 1; Fig. 1). Obese adolescents with HI and HI + IGT had higher fasting insulin, insulin AUC and HOMA-IR, and lower Matsuda Index, than lean adolescents and obese adolescents with NI. Fasting glucose was significantly higher in obese adolescents with IGT than in the lean control group, and their glucose AUC (obese HI + IGT) was significantly higher than in the three other groups (lean; obese NI; obese HI).

**Table 1:** Descriptive statistics on the study population

	Lean, NI	Obese, NI	Obese, HI	Obese, HI + IGT	Group^1^
n (male/female)	21 (13/8)	18 (10/8)	20 (14/6)	23 (13/10)	0.784
Puberty (n; male/female)					0.471
Pre-pubertal	7	4	4	2	
Pubertal	6	9	9	10	
Post-pubertal	8	5	7	11	
Age (years)	13.9 ± 0.49	13.1 ± 0.53	14.2 ± 0.5	14.5 ± 0.47	0.208
BMI (kg/m^2^)	18.5 ± 1.2^a^	31.8 ± 1.29^b^	35.8 ± 1.23^b,c^	36.5 ± 1.14^c^	< 0.0001
BMI-SDS	−0.3 ± 0.15^a^	2.9 ± 0.16^b^	3.2 ± 0.15^b^	3.2 ± 0.14^b^	< 0.0001
Fasting insulin (mIU/L)	8.4 ± 2.19^a^	15.9 ± 2.36^a^	29.2 ± 2.24^b^	28.8 ± 2.08^b^	< 0.0001
AUC insulin (mIU/L × min)	6115 ± 1138^a^	8873 ± 1229^a^	18,680 ± 1166^b^	16,619 ± 1085^b^	< 0.0001
Fasting glucose (mM)	5.4 ± 0.08^a^	5.5 ± 0.09	5.6 ± 0.08	5.8 ± 0.08^b^	0.030
AUC glucose (mM × min)	802 ± 19.2^a^	826 ± 20.3^a^	872 ± 19.2^a^	983 ± 17.9^b^	< 0.0001
HOMA-IR	2.05 ± 0.56^a^	3.92 ± 0.6^a^	7.24 ± 0.57^b^	7.4 ± 0.53^b^	< 0.001
Matsuda Index	5.3 ± 0.31^a^	3.5 ± 0.32^b^	1.7 ± 0.30^c^	1.9 ± 0.28^c^	< 0.0001

*AUC* area under the curve; *BMI* body mass index, *HI* high insulin; *HOMA-IR* homeostasis assessment of insulin resistance; *IGT* impaired glucose tolerance; *NI* normal insulin; *SDS* standard deviation score; *SEM* standard error of the mean

Values are mean ± SEM

Values within rows with different superscript letters are significantly different

^1^Calculated by Fisher’s exact test (categorical data) or ANOVA with Bonferroni post-hoc test (numerical data)

### Metabolic differences between the groups

Using the univariate statistical approach (mixed model with time, group and their interaction (time × group) as fixed factors, first step in univariate analysis), followed by BH correction for multiple testing, amino acids (AA) such as isoleucine, leucine, valine, tyrosine, phenylalanine, glutamate, serine, alanine, an AA metabolite (2-oxoisocaproate), dimethylsulfone, acetate and o-acetylcarnitine were identified as discriminative (significant group effect). Myo-inositol also showed a significant time × group interaction after BH correction. In the multivariate statistical approach comparing metabolic profiles between groups at each time point, PLS-DA models at 15, 30, 60, 90 and 120 min were significant, with cross-validated ANOVA *P-*values < 0.05 (Online Resource 3). All above-mentioned metabolites, except for o-acetylcarnitine, were confirmed as discriminative by the multivariate statistical approach. In addition, myo-inositol (60, 90, 120 min), pyruvate (60 min), lysine (120 min) and glycine (30, 60, 90, 120 min) were identified as discriminative by the multivariate approach.

All metabolites found to be discriminative in univariate and multivariate statistical analyses were further investigated using a mixed model with time, insulin response, obesity, glucose response and their interactions as fixed factors (fifth step in univariate analysis). The results are presented in Online Resource 4. Isoleucine, leucine, valine, tyrosine, serine, dimethylsulfone, lysine, phenylalanine and 2-oxoisocaproate showed a significant insulin response effect (insulin response NI vs. HI as fixed factor) (*P* < 0.05). O-acetylcarnitine, glutamate, alanine and acetate showed a significant obesity effect (obesity status lean vs. obese as fixed factor) (*P* < 0.05). Myo-inositol showed a significant time × obesity interaction (*P* < 0.05), while for pyruvate, time × obesity interaction tended to be significant (*P =* 0.0551).

Statistical analysis and plots of changes over time revealed that the discriminative metabolites could be divided into two groups: (1) metabolites discriminating between adolescents with NI vs. HI (groups 1–2 vs. groups 3–4) and (2) metabolites discriminating between lean and obese adolescents (group 1 vs. groups 2–4).

#### Metabolites discriminating between adolescents with NI vs. HI (groups 1–2 vs. groups 3–4)

**Fig. 2:**
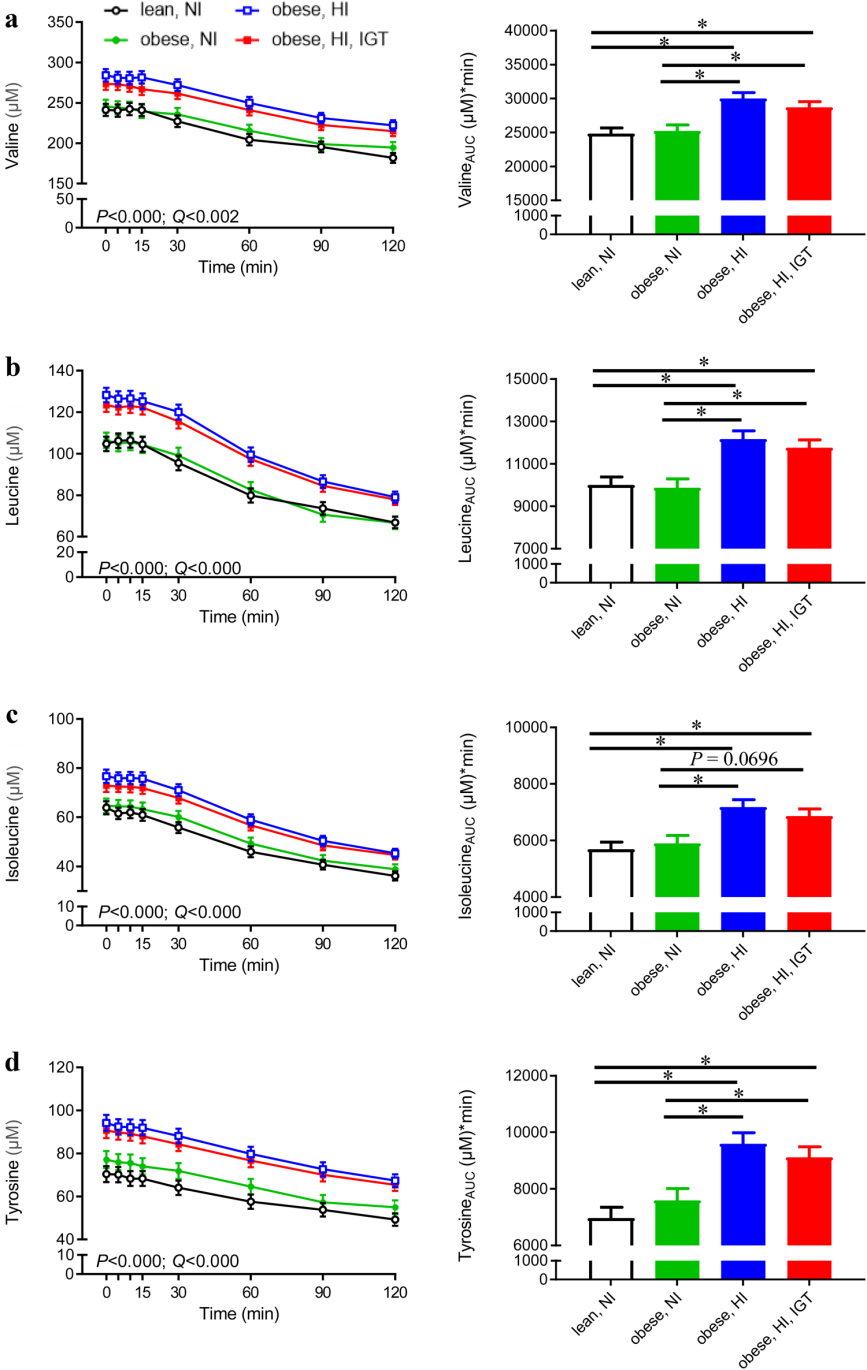
Plasma levels during an oral glucose tolerance test (OGTT; left panel) and area under the curve (AUC; right panel) of **a** valine, **b** leucine, **c** isoleucine and **d** tyrosine in lean adolescents with normal insulin (NI; black, n = 21), adolescents with obesity and NI (green, n = 18), adolescents with obesity and high insulin (HI; blue, n = 20), and adolescents with obesity and HI in combination with impaired glucose tolerance (IGT; red, n = 23). *P*-values for group effect (n = 4, mixed model) and corresponding Benjamini-Hochberg-adjusted *P*-values (*Q*-values) are presented. All metabolites also showed a significant insulin response effect (NI vs. HI) (*P* < 0.05) (Color figure online)

Concentrations of the BCAAs valine, leucine and isoleucine and the AAA tyrosine were lower in adolescents with NI secretion (lean and obese, groups 1–2) than in obese adolescents with HI secretion in response to the OGTT (with and without IGT, groups 3–4) (Fig. 2, left panel). The concentrations were significantly different at almost all sampling time points (for *P*-values, see Online Resource 5). The AUCs of valine, leucine, isoleucine and tyrosine were consistently and significantly lower in lean and obese adolescents with NI (groups 1–2) than in obese adolescents with HI (groups 3–4) (Fig. 2, right panel).

**Fig. 3:**
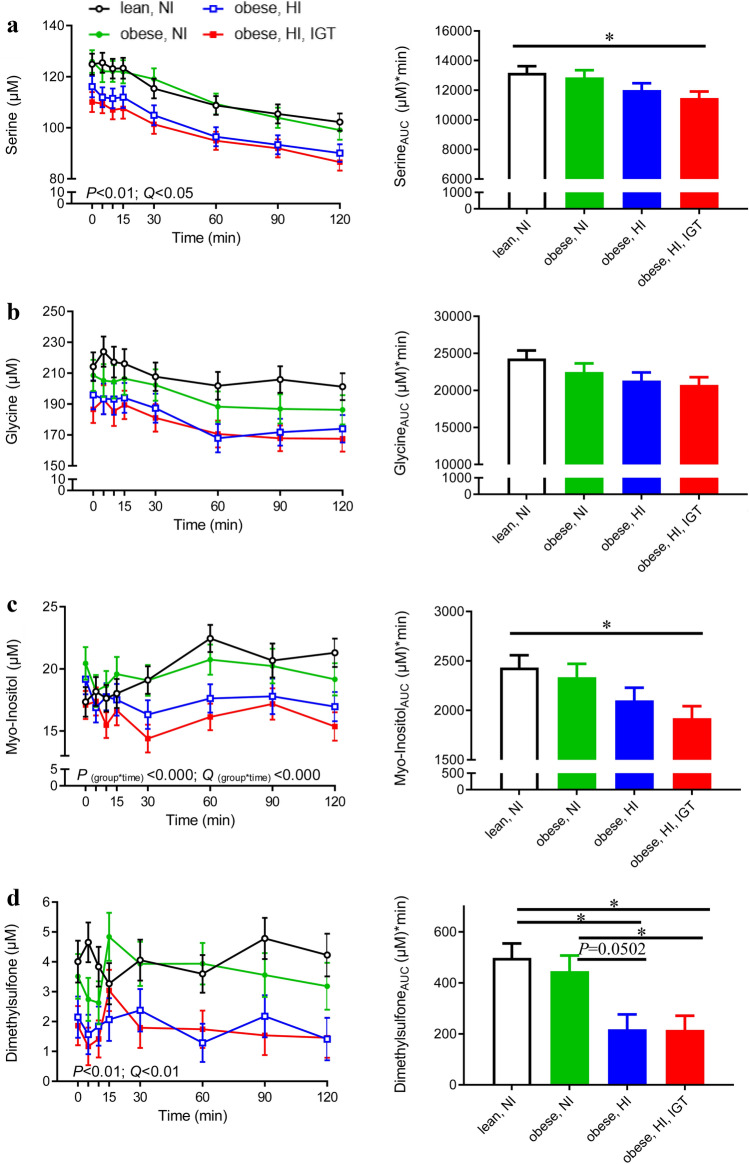
Plasma levels during an oral glucose tolerance test (OGTT; left panel) and area under the curve (AUC; right panel) of **a** serine, **b** glycine, **c** myo-inositol and **d** dimethylsulfone in lean adolescents with normal insulin (NI; black, n = 21), adolescents with obesity and NI (green, n = 18), adolescents with obesity and high insulin (HI; blue, n = 20), and adolescents with obesity and HI in combination with impaired glucose tolerance (IGT; red, n = 23). *P*-values for group effect (n = 4, mixed model), time × group interaction and corresponding Benjamini-Hochberg-adjusted *P*-values (*Q*-values) are presented for metabolites identified as discriminative via the univariate statistical approach (serine, dimethylsulfone and myo-inositol). Serine and dimethylsulfone also showed a significant insulin response effect (NI vs. HI) (*P* < 0.05). Glycine was identified as discriminative *via* the multivariate statistical approach and significant models were obtained at 30, 60, 90 and 120 min (Color figure online)

Phenylalanine, lysine and 2-oxoisocaproic acid levels and their AUCs showed similar patterns to the above-mentioned metabolites (Online Resource 6) and followed the pattern of being lower in lean and obese adolescents with NI than in obese adolescents with HI (with and without IGT). In contrast, serine, glycine, myo-inositol and dimethylsulfone were inversely associated with insulin concentration (Fig. 3). Differences were most pronounced between lean adolescents and obese adolescents with HI + IGT, for which serine concentrations differed at all time points and glycine reached statistical significance 10, 90 and 120 min after the OGTT (for *P*-values see Online Resource 5). Differences between the groups in concentrations of myo-inositol and dimethylsulfone were most pronounced postprandially from 30 to 120 min after glucose ingestion (Fig. 3c, d, left panel). In addition, myo-inositol showed a significant group × time effect and obesity × time effect, reflected in decreasing myo-inositol concentrations in response to the OGTT in obese adolescents with HI and HI + IGT (− 8% and − 6% at 60 min, respectively), almost constant myo-inositol concentrations in obese adolescents with NI (+ 1.5% at 60 min) and increasing myo-inositol levels in lean adolescents (+ 29% at 60 min).

#### Metabolites discriminating between lean and obese adolescents (group 1 vs. groups 2–4)

**Fig. 4:**
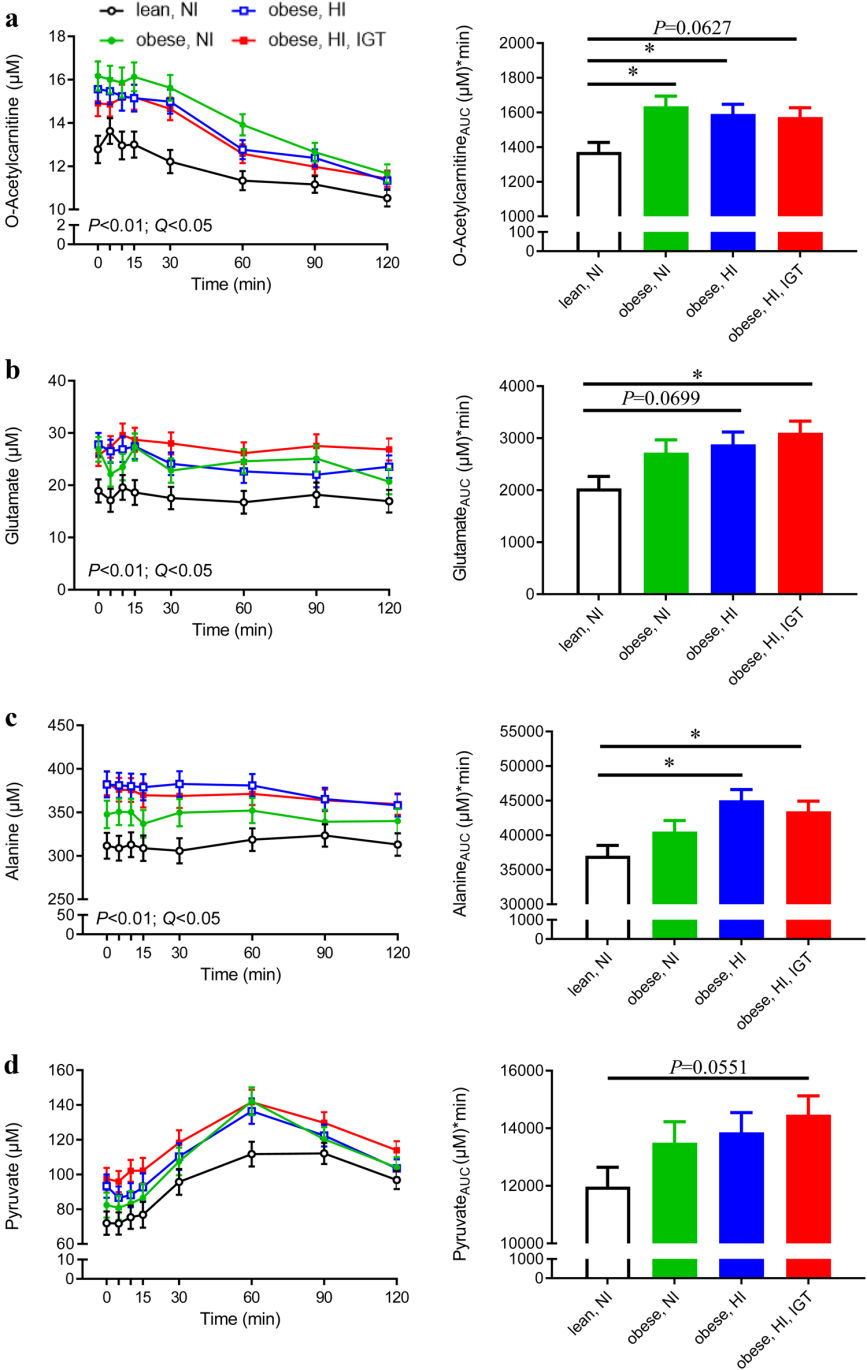
Plasma levels during an oral glucose tolerance test (OGTT; left panel) and area under the curve (AUC; right panel) of **a** o-acetylcholine, **b** glutamate, **c** alanine and **d** pyruvate in lean adolescents with normal insulin (NI; black, n = 21), adolescents with obesity and NI (green, n = 18), adolescents with obesity and high insulin (HI; blue, n = 20), and adolescents with obesity and HI in combination with impaired glucose tolerance (IGT; red, n = 23). *P*-values for group (n = 4, mixed model) and corresponding Benjamini-Hochberg-adjusted *P*-values (*Q*-values) are presented for metabolites identified as discriminative via the univariate statistical approach (o-acetylcarnitine, glutamate and alanine). O-acetylcarnitine, glutamate and alanine also showed a significant obesity effect (lean vs. obese) (*P* < 0.05). Pyruvate was identified as discriminative *via* the multivariate statistical approach and a significant model was obtained at 60 min. Pyruvate also showed a close to significant time × obesity interaction (*P =* 0.0551) (Color figure online)

**Fig. 5:**
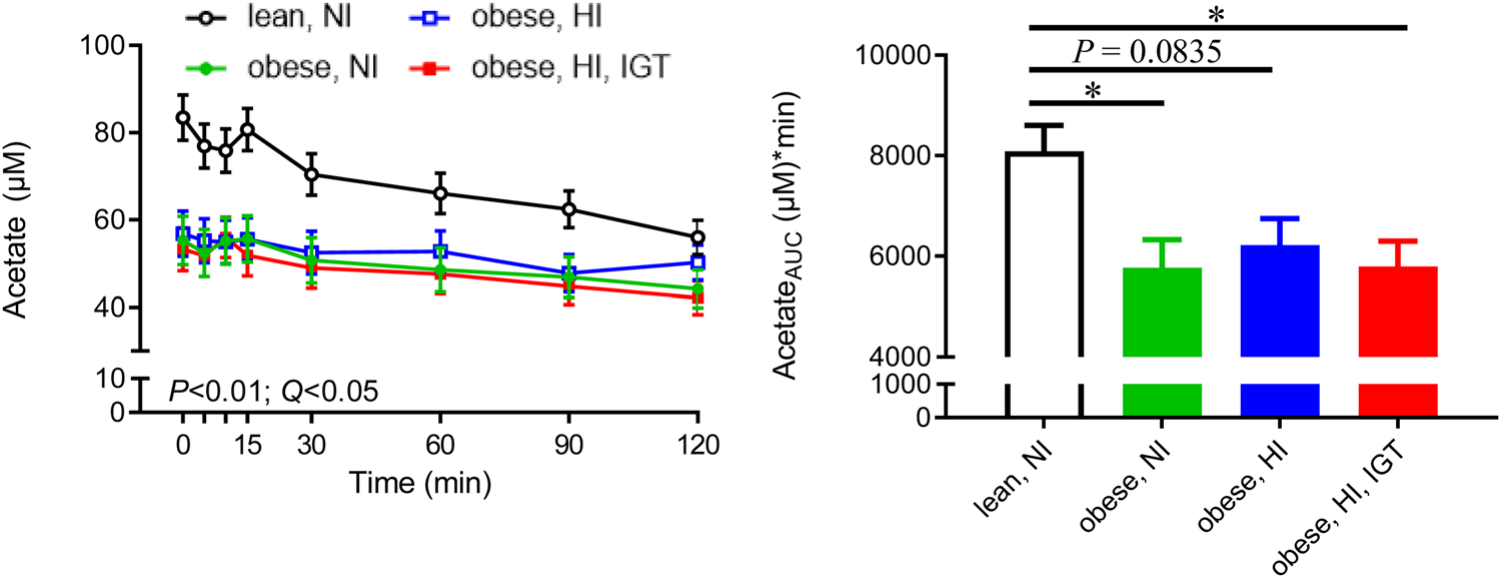
Plasma levels during an oral glucose tolerance test (OGTT; left panel) and area under the curve (AUC; right panel) of acetone in lean adolescents with normal insulin (NI; black, n = 21), adolescents with obesity and NI (green, n = 18), adolescents with obesity and high insulin (HI; blue, n = 20), and adolescents with obesity and HI in combination with impaired glucose tolerance (IGT; red, n = 22). *P*-values for group (n = 4, mixed model) and corresponding Benjamini-Hochberg-adjusted *P*-values (*Q*-values) are presented. Acetate also showed a significant obesity effect (lean vs. obese) (*P* < 0.05) (Color figure online)

O-acetylcarnitine, glutamate, alanine and pyruvate concentrations were higher, or showed a tendency to be higher, in obese individuals (groups 2–4) than in lean adolescents (group 1) (Fig. 4). Differences in o-acetylcarnitine (Fig. 4a) were most pronounced between lean adolescents and obese adolescents with NI (for *P*-values, see Online Resource 5). Differences in glutamate, alanine and pyruvate were most pronounced between lean adolescents and adolescents with HI + IGT (Fig. 4b–d; for *P*-values, see Online Resource 5). Acetate was inversely associated with obesity, as reflected in higher acetate concentrations in lean compared with obese adolescents (Fig. 5).

#### Adjustment for puberty and sex

The distribution of pubertal status across the four groups of lean and obese adolescents with NI or HI was comparable (Table 1) and sex ratio was similar between the groups. When univariate statistical analyses (mixed model with four groups, first step in univariate analysis) were re-estimated by adjusting for pubertal status and sex, all metabolites previously identified as discriminative (without adjustment) were confirmed even after secondary correction for multiple testing, except for o-acetylcarnitine, which showed significant differences between the groups after adjustment for puberty and sex (*P* = 0.0166), but only tended to be significant after correction for multiple testing (Q = 0.0581; Online Resource 7). When the model was corrected for BMI in addition to puberty and sex, among the metabolites which were discriminating between adolescents with NI vs. HI, valine, leucine, tyrosine, serine, dimethylsulfone and lysine showed a significant group effect (*P* < 0.05), and myo-inositol, phenylalanine and 2-oxoisocaproate showed a significant time × group interaction (*P* < 0.05).

## Discussion

In the present study, two groups of metabolites were identified. The first group discriminated adolescents with NI (lean and obese) from those with HI (obese with and without IGT) and comprised three BCAAs (valine, isoleucine, and leucine), a catabolic intermediate of leucine (2-oxoisocaproate), tyrosine, phenylalanine, lysine, serine, myo-inositol and dimethylsulfone. The second group discriminated lean from obese adolescents and comprised o-acetylcarnitine, pyruvate, glutamate, alanine and acetate.

### Metabolites discriminating adolescents with normal insulin levels from those with high insulin

BCAAs are suggested to be predictive of future T2DM (Wang et al. 2011), and are associated with poor metabolic health (Badoud et al. 2014; Newgard et al. 2009). Our findings that BCAAs are higher in obese adolescents with hyperinsulinaemia/IR are consistent with previous studies in adolescents showing higher BCAAs in obese compared with lean subjects (Butte et al. 2015; Perng et al. 2014; Short et al. 2019) and positive associations between BCAAs and IR (even) after adjustment for BMI (Suzuki et al. 2019; Tricò et al. 2017; Zhang et al. 2019). However, data on BCAAs and metabolic health in adolescents are inconsistent (Farook et al. 2015; Michaliszyn et al. 2012; Mihalik et al. 2012; Wahl et al. 2012). These inconsistencies may partly be related to the characteristics of the study population. For example, in a study where no differences in BCAAs were found between lean and obese subjects, the two groups had comparable HOMA-IR (Farook et al. 2015). Because of the prevalence of IR in obesity, in most of the studies mentioned above there was coherence between IR and obesity in the study population. The number of studies discriminating between metabolically healthy and unhealthy obese adolescents is limited.

Mastrangelo et al. (2016) compared the metabolic profile in obese children with and without IR and found higher BCAA levels in obese children with IR, as also found in the present study. However, their study did not include a normal-weight lean control group. Our findings are also corroborated by studies on adults showing higher BCAAs in normal-weight IR individuals compared with normal-weight insulin-sensitive participants (Tai et al. 2010), and by reports of similar BCAA concentrations in lean and metabolically healthy obese individuals, but increased levels in metabolically unhealthy, centrally obese participants (Gao et al. 2016).

Studies using Mendelian randomisation analyses to investigate the causal relationship between IR and BCAAs provide genetic evidence that IR can lead to elevated circulating BCAA levels (Mahendran et al. 2017; Wang et al. 2017) and indicate a causal pathway from adiposity, *via* IR and BCAAs, to diabetes (Wang et al. 2017).

Circulating BCAA levels are determined by their rates of appearance (i.e. dietary intake and protein degradation) and disappearance (i.e. oxidative catabolism and non-oxidative disposal) (Lynch and Adams 2014). It has been shown that, despite similar dietary protein intake, non-obese IR individuals with higher circulating BCAAs have higher leucine flux and weaker leucine oxidation relative to total leucine flux than non-obese, insulin-sensitive individuals (Tan et al. 2018). This may be explained by findings that insulin action enhances the activity of branched-chain α-keto acid dehydrogenase, the rate-limiting enzyme in catabolism of BCAAs (Shin et al. 2014).

Hypotheses have also emerged of a causal effect of BCAAs in IR *via* (i) persistent activation of the mammalian target of the rapamycin complex 1 (mTORC1) pathway (Newgard 2012; Newgard et al. 2009), and (ii) impaired BCAA metabolism, leading to accumulation of toxic intermediates, which in turn causes mitochondrial stress/dysfunction and impairs insulin action (Newgard 2012; Newgard et al. 2009). We found higher levels of the catabolic intermediate of leucine, 2-oxoisocaproate, in obese adolescents with HI, which may be related to the latter hypothesis. However, in a previous study supplementation with BCAAs did not affect plasma BCAAs concentrations in obese pre-diabetic individuals and tended to improve glucose metabolism (Woo et al. 2019). Reduced BCAAs intake in T2DM individuals has been found to reduce plasma BCAAs, despite no effect on insulin sensitivity under clamp conditions (Karusheva et al. 2019). Phenylalanine and tyrosine showed similar patterns to BCAAs in the present study. Both amino acids have been reported consistently in adults with obesity (Newgard et al. 2009), IR (Tai et al. 2010) and T2DM (Ha et al. 2012).

In contrast to BCAAs and AAAs, serine and glycine were present in lower concentrations in obese adolescents with HI or HI + IGT. Lower serine and glycine levels have also been reported in adolescents with obesity (Butte et al. 2015) and T2DM (Mihalik et al. 2012). Glycine has even been associated with a decreased risk of IGT (Wang-Sattler et al. 2012) and/or T2DM (Floegel et al. 2013; Wang-Sattler et al. 2012) in adults. Serine and glycine can be synthesised *de novo* by the human body and the contribution of endogenous synthesis to the pool of serine is almost 94% (Reeds 2000). Therefore, differences in serine concentrations may be due to differences in endogenous serine synthesis between NI (groups 1–2) and HI (groups 3–4) individuals. It has been shown consistently that expression of the enzyme phosphoserine aminotransferase 1, involved in serine synthesis, is reduced in high-fat diet-induced diabetic mice (Yu et al. 2015). Alternatively, metabolic consumption of serine (e.g. for gluconeogenesis) and glycine (e.g. glutathione production) (Sekhar et al. 2011) may be higher under conditions of hyperinsulinaemia/IR.

To the best of our knowledge, ours is the first study to demonstrate that myo-inositol and dimethylsulfone are discriminative between adolescents with HI (groups 3–4) and NI (groups 1–2). Myo-inositol can be synthesised *de novo* from glucose. Intriguingly, differences in myo-inositol between the groups were more apparent at 30 and 60 min after OGTT, when glucose and insulin levels were also significantly elevated compared with baseline. In addition, myo-inositol showed significant time × group and time × obesity interactions. The increase in myo-inositol concentration in lean adolescents during OGTT may be related to its higher *de novo* synthesis from glucose (Croze and Soulage 2013). In addition, studies on diabetic individuals have observed higher urinary excretion of myo-inositol (Kennington et al. 1990) and lower levels of inositol phosphoglycans in muscle biopsies (Croze and Soulage 2013). Dimethylsulfone, an organic sulphur compound, was present in lower concentrations in obese adolescents with HI and HI + IGT (groups 3–4). In the human body, dimethylsulfone can originate from dietary sources, from intestinal bacterial metabolism and from endogenous methane thiol metabolism (Engelke et al. 2005). However, further investigations are needed to identify underlying mechanisms of lower myo-inositol and dimethylsulfone responses in conditions of hyperinsulinaemia/IR.

### Metabolites discriminating lean and obese adolescents

Acetylcarnitine levels (C2) were higher in obese than in lean adolescents. Interestingly, these differences were most pronounced between lean adolescents and obese adolescents with NI. Acylcarnitines in general reflect fatty acid flux through β-oxidation. In response to an OGTT (Zhao et al. 2009) or a food challenge (Krug et al. 2012; Shrestha et al. 2017), acylcarnitines decrease due to the switch from β-oxidation to glycolysis. This decrease was observed in all four groups in the present study. It has previously been suggested that acetylcarnitine is released to plasma when mitochondrial acetyl-CoA concentrations exceed their entry capacity into the tricarboxylic acid (TCA) cycle. Acetyl-CoA, if not exported, can otherwise inhibit pyruvate dehydrogenase (PDH), a key regulatory enzyme connecting glycolysis to glucose oxidation (Muoio et al. 2012). The differences in acetylcarnitine concentrations that we observed between lean and obese adolescents may therefore indicate a mismatch between β-oxidation and TCA-cycle activity in obese individuals. The suggestion of reduced PDH activity in obese adolescents is in line with the higher pyruvate levels (PDH substrate) in obese compared with lean adolescents in the present study. Alanine can be synthesised from pyruvate (correlation between the two metabolites: r = 0.615, *P* < 0.0001) after receiving an amino group from glutamate. Both glutamate and alanine were higher in obese adolescents. Consistent with the hypothesis of reduced PDH activity in obese adolescents, data from mechanistic studies show increased expression of PDH kinase 4 mRNA in diabetes (Kulkarni et al. 2012) and decreased PDH phosphatase activity in obesity (LeBlanc et al. 2008), both leading to reduced PDH activity. Our observation of increased pyruvate, alanine and glutamate plasma concentrations in obese adolescents is in agreement with findings in studies comparing lean and obese adults (Newgard et al. 2009). Glutamate concentrations are reported to be similar between metabolically healthy and unhealthy obese adults, but significantly lower in lean control adults (Badoud et al. 2014), as also found for adolescents in the present study.

Another metabolite distinguishing between lean and obese adolescents was acetate, which showed higher concentrations in lean individuals and was therefore inversely related to BMI (in contrast to pyruvate, alanine and glutamate). Acetate can be derived from exogenous production by the gut microbiota or from endogenous metabolism of amino acids, carbohydrates and fatty acids. In humans, endogenous metabolism is suggested to be the major source of plasma acetate (Piloquet et al. 2003). In the present study, acetate was inversely correlated to alanine, pyruvate and lactate (r = − 0.481, r = − 0.346, r = − 0.349 respectively, *P* < 0.01 for all). Thus, higher acetate levels in healthy lean adolescents might originate from higher acetate production from the 3-carbon metabolites (i.e. pyruvate). This is consistent with the hypothesis of reduced PDH activity in obesity discussed above.

The main limitations of the present study were use of only a targeted metabolomics approach, low number of participants, using only IR surrogates, assessing pubertal stage based on sex hormones and not including individuals with IFG and T2DM. In addition, group 2 (obese, NI) was not as insulin-sensitive as the lean group (group 1), even though its Matsuda Index was above the threshold (2.5) for insulin sensitivity (Masuda Index = 3.5 ± 0.32). Larger studies in which additional groups are included, i.e. overweight and IR adolescents with different levels of IR, are warranted.

## Conclusions

After selection and assignment of adolescents into groups, defined based on BMI, insulin secretory patterns and glucose tolerance, the present study revealed clusters of metabolites associated either with insulin response (hyperinsulinaemia/IR), or with obesity. Our data suggest that BCAA and AAA are associated with hyperinsulinaemia/IR in obese adolescents. Our data also suggest that o-acetylcarnitine, glutamate, pyruvate, alanine and acetate are associated with obesity, indicating shifts in fat and glucose metabolism. Whether BCAA and AAA are mechanistically involved, or markers of poor metabolic health, and whether they can be used as a marker of hyperinsulinaemia/IR in adolescents warrants further investigation. Our findings shed light on these metabolic alterations as possible hallmarks of the predisposing conditions (i.e. obesity and hyperinsulinaemia/IR) on the pathway toward further complications and eventually development of T2DM. The biological consequences of the metabolic alterations associated with obesity are of great importance, as obesity is the most important risk factor for IR and further complications such as T2DM.

## Supplementary Information

Below is the link to the electronic supplementary material.

Supplementary material 1 (PDF 24.6 kb)

Supplementary material 2 (PDF 121.0 kb)

Supplementary material 3 (PDF 180.8 kb)

Supplementary material 4 (PDF 25.7 kb)

Supplementary material 5 (PDF 366.1 kb)

Supplementary material 6 (PDF 65.4 kb)

Supplementary material 7 (PDF 159.6 kb)

## Data Availability

The data used in the present study are available to the Editorial board for evaluating the findings presented. Metabolomics data are also available from the corresponding authors upon reasonable request.
