# Supplementary material for: Metabolomics analysis reveals altered metabolites in lean compared with obese adolescents and additional metabolic shifts associated with hyperinsulinaemia and insulin resistance in obese adolescents: a cross-sectional study
Source: Metabolomics. 2021 Jan 12;17(1):11. doi: 10.1007/s11306-020-01759-y (PMC7803706; doi:10.1007/s11306-020-01759-y)
Supplement: Supplementary file 1 — Supplementary material 1 (PDF 24.6 kb) [file 11306_2020_1759_MOESM1_ESM.pdf]

**Metabolomics analysis reveals altered metabolites in lean compared with obese adolescents and additional metabolic shifts associated with hyperinsulinaemia and insulin resistance in obese adolescents: a cross-sectional study**

Elisabeth Müllner, Hanna E. Röhnisch, Claudia von Brömssen, Ali A. Moazzami*
*Corresponding author: Ali.Moazzami@slu.se; Department of Molecular Sciences, Swedish University of Agricultural Sciences, Uppsala, Sweden

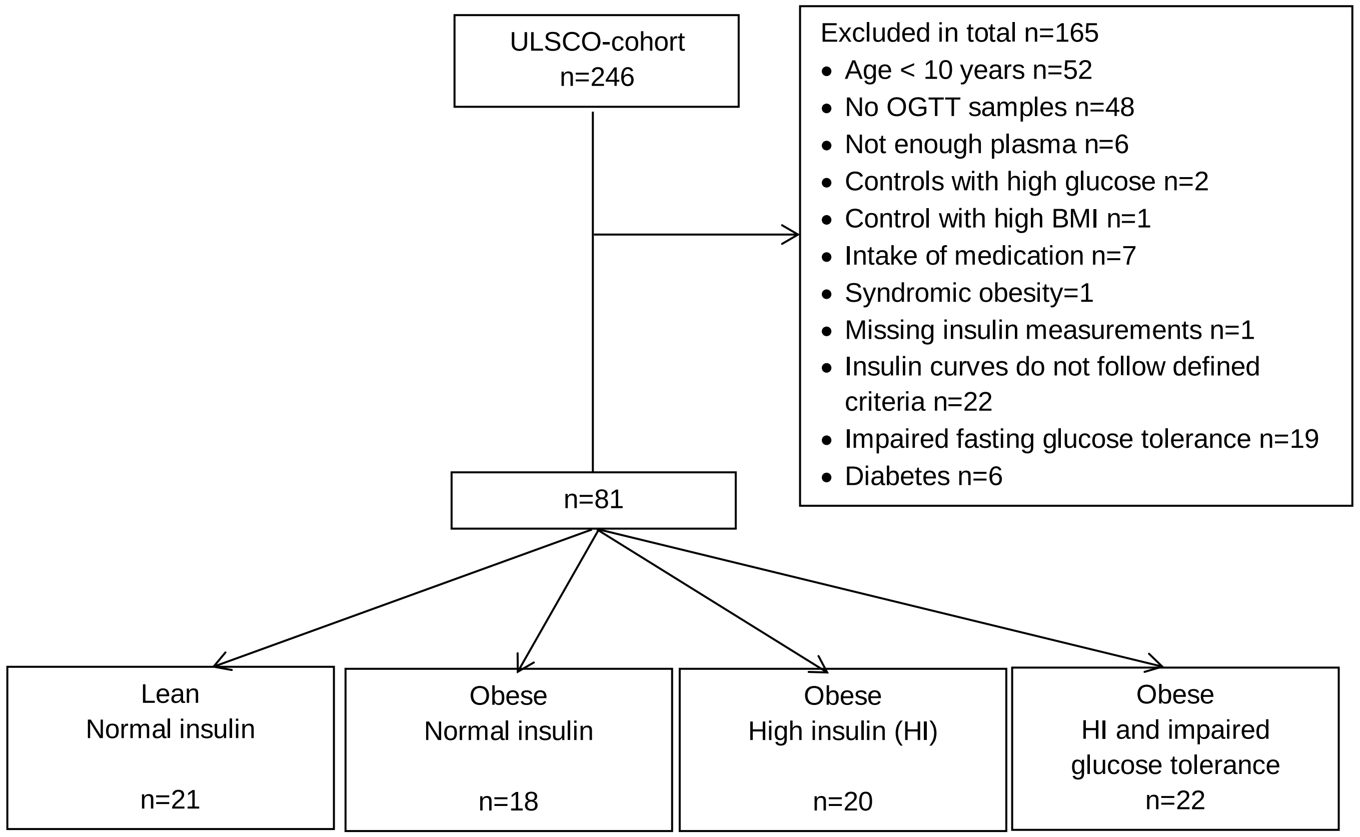


**Online Resource 1:** Flow chart illustrating the in- and exclusion of adolescents in the metabolomics study.
