# Supplementary material for: Metabolomics analysis reveals altered metabolites in lean compared with obese adolescents and additional metabolic shifts associated with hyperinsulinaemia and insulin resistance in obese adolescents: a cross-sectional study
Source: Metabolomics. 2021 Jan 12;17(1):11. doi: 10.1007/s11306-020-01759-y (PMC7803706; doi:10.1007/s11306-020-01759-y)
Supplement: Supplementary file 2 — Supplementary material 2 (PDF 121.0 kb) [file 11306_2020_1759_MOESM2_ESM.pdf]

Metabolomics analysis reveals altered metabolites in lean compared with obese adolescents and additional metabolic shifts associated with hyperinsulinaemia and insulin resistance in obese adolescents: a cross-sectional study

Elisabeth Müllner, Hanna E. Röhnisch, Claudia von Brömssen, Ali A. Moazzami*
*Corresponding author: Ali.Moazzami@slu.se; Department of Molecular Sciences, Swedish University of Agricultural Sciences, Uppsala, Sweden

Online Resource 2

Anthropometry

Body height was measured by a stadiometer (Ulmer stadiometer, Busse, Elchingen, Germany) and body weight by a digital scale (SECA model 704, Hamburg, Germany). BMI was calculated as kg/m^2. Sex- and age-independent BMI standard deviation score (SDS) was calculated using the WHO 2006-2007 growth reference. Pubertal stage was assessed using sex-specific cut-off points for sex hormones and growth charts for height.

Clinical assessment

Glucose (Abbott Architect, Abbott Diagnostics, Lake Forest, Ill) and insulin (Cobas E602, Roche Diagnostics, Indianapolis, IN) analyses were performed at the University Hospital, Uppsala, Sweden. Insulin data from four control subjects were missing and were therefore analysed by enzyme linked immunosorbent assay (ELISA; Mercodia). The HOMA-IR was calculated as fasting insulin (µIU/mL) × fasting glucose (mmol/mL)/22.5 (Matthews *et al.*, 1985). Matsuda Index was calculated as described previously (Matsuda and DeFrozo, 1999).

Sample and data retrieval

Plasma samples were retrieved from ULSCO cohort (Forslund *et al.*, 2014). Insulin and glucose concentrations, gender, status of puberty, age and BMI were retrieved from the database of ULSCO cohort (Forslund *et al.*, 2014).

Metabolic profiling

Ethylenediaminetetraacetic acid (EDTA) plasma samples were used for metabolomics analyses. Sample preparation was performed based on a previously described protocol (Röhnisch *et al.*, 2018; Tiziani *et al.*, 2008). In brief, Nanosep centrifugal filters with 3-kDa cut-off (Pall Life Science, Port Washington, NY) were washed 8 times with 500 µL water at 2,000 g and 36°C. Then 60 µL of plasma sample were filtered at 10,000 g, 4°C. For quantification of metabolites, 40 µL of plasma filtrate were mixed with 50 µL phosphate buffer (0.4 mol/L, pH 7.0), 15 µL D_2O, 55 µL Millipore water and 10 µL sodium-3-(trimethlsilyl)-2,2,3,3,-tetradeuteriopropionate (TSP, 5.8 mmol/L) (Cambridge Isotope Laboratories, Andover, MA) as an internal standard. Analyses were performed on a Bruker spectrometer operating at 600 MHz equipped with a cryogenically cooled probe and autosampler. The nuclear magnetic resonance (^1H NMR) spectra were obtained using the zgesgp pulse sequence (Bruker

Spectrospin Ltd) at 25°C with 512 scans at 65,356 data points over a spectral width of 17,942.58 Hz (acquisition time: 1.83 sec, relaxation delay 4 sec) (Hwang and Shaka, 1995; Röhnisch *et al.*, 2018). Forty-nine metabolites were identified based on previous literature (Nagana Gowda *et al.*, 2015), search in human metabolome database (HMDB), spiking with authentic standards and commercial library (NMR Suite Professional Software package, version 7.5, ChenomX Inc., Edmonton, Canada) as previously described (Röhnisch *et al.*, 2018). For the list of identified metabolites and their HMDB ID (Metabolomics Standard Initiative (MSI) identification, at least, level 2) see Online Resource 7.

The concentrations of the identified metabolites were calculated using an automated quantification algorithm (AQuA) capable of accounting for interfering signals (Röhnisch *et al.*, 2018). AQuA was modified by inclusion of EDTA and its calcium and magnesium complexes (Barton *et al.*, 2010) in the metabolite library, in order to account for interferences of EDTA signals with signals from other metabolites.

References

Barton, R.H., Waterman, D., Bonner, F.W., Holmes, E., Clarke, R., the, P.C., Nicholson, J.K. and Lindon, J.C. (2010) The influence of EDTA and citrateanticoagulant addition to human plasma on information recovery from NMR-based metabolic profiling studies. *Molecular BioSystems* **6,** 215-224.

Forslund, A., Staaf, J., Kullberg, J., Ciba, I., Dahlbom, M. and Bergsten, P. (2014) Uppsala Longitudinal Study of Childhood Obesity: Protocol Description. *Pediatrics*.

Hwang, T.L. and Shaka, A.J. (1995) Water Suppression That Works - Excitation Sculpting Using Arbitrary Wave-Forms and Pulsed-Field Gradients. *Journal of Magnetic Resonance Series A* **112,** 275-279.

Matsuda, M. and DeFrozo, R.A. (1999) Insulin sensitivity indices obtained from oral glucose tolerance testing: comparison with the euglycemic insulin clamp. *Diabetes Care* **22,** 1462-1470.

Matthews, D.R., Hosker, J.P. and Rudenski, A.S. (1985) Homeostasis model assessment: Insulin resistance and β-cell function from fasting plasma glucose and insulin concentrations in man. *Diabetologia* **28,** 412-419.

Nagana Gowda, G.A., Gowda, Y.N. and Raftery, D. (2015) Expanding the limits of human blood metabolite quantitation using NMR spectroscopy. *Anal Chem* **87,** 706-15.

Röhnisch, H.E., Eriksson, J., Müllner, E., Agback, P., Sandström, C. and Moazzami, A.A. (2018) AQuA: An Automated Quantification Algorithm for High-Throughput NMR-Based Metabolomics and Its Application in Human Plasma. *Analytical Chemistry* **90,** 2095-2102.

Tiziani, S., Emwas, A.H., Lodi, A., Ludwig, C., Bunce, C.M., Viant, M.R. and Gunther, U.L. (2008) Optimized metabolite extraction from blood serum for 1H nuclear magnetic resonance spectroscopy. *Anal Biochem* **377,** 16-23.
