# Supplementary material for: Metabolomics analysis reveals altered metabolites in lean compared with obese adolescents and additional metabolic shifts associated with hyperinsulinaemia and insulin resistance in obese adolescents: a cross-sectional study
Source: Metabolomics. 2021 Jan 12;17(1):11. doi: 10.1007/s11306-020-01759-y (PMC7803706; doi:10.1007/s11306-020-01759-y)
Supplement: Supplementary file 3 — Supplementary material 3 (PDF 180.8 kb) [file 11306_2020_1759_MOESM3_ESM.pdf]

Metabolomics analysis reveals altered metabolites in lean compared with obese adolescents and additional metabolic shifts associated with hyperinsulinaemia and insulin resistance in obese adolescents: a cross-sectional study

Elisabeth Müllner, Hanna E. Röhnisch, Claudia von Brömssen, Ali A. Moazzami*
*Corresponding author: Ali.Moazzami@slu.se; Department of Molecular Sciences, Swedish University of Agricultural Sciences, Uppsala, Sweden

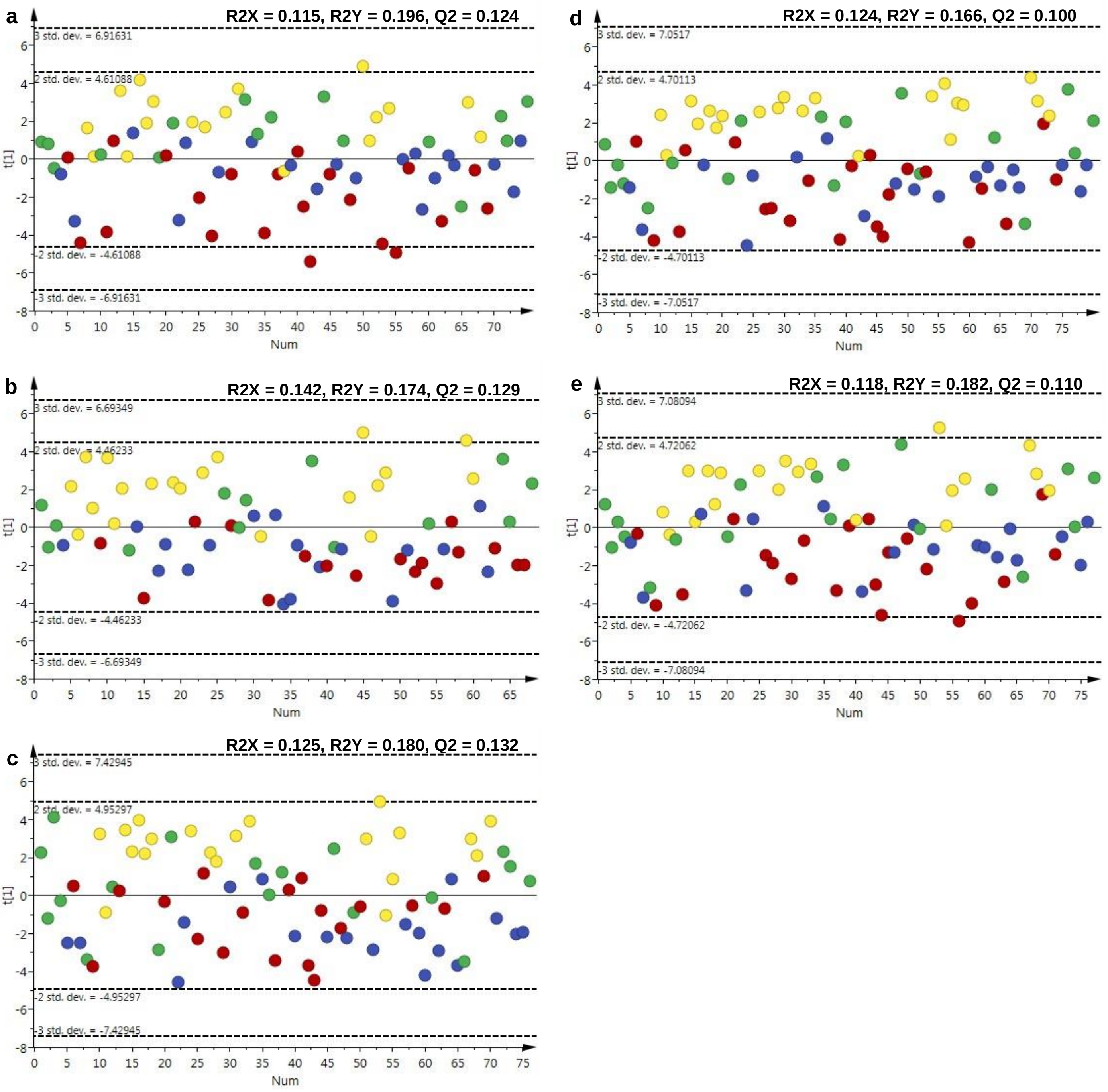

Online Resource 3: Partial least square discriminant analysis (PLS-DA) for healthy lean controls with normal insulin levels (yellow), adolescents with obesity and normal insulin levels (green), obesity and high insulin (red) and obesity, high insulin and impaired glucose tolerance (blue) at time point (a) 15 min, (b) 30 min, (c) 60 min, (d) 90 min and (e) 120 min. All models were significant with a *P*-value from the cross-validated ANOVA <0.05.
