# Supplementary material for: Metabolomics analysis reveals altered metabolites in lean compared with obese adolescents and additional metabolic shifts associated with hyperinsulinaemia and insulin resistance in obese adolescents: a cross-sectional study
Source: Metabolomics. 2021 Jan 12;17(1):11. doi: 10.1007/s11306-020-01759-y (PMC7803706; doi:10.1007/s11306-020-01759-y)
Supplement: Supplementary file 4 — Supplementary material 4 (PDF 25.7 kb) [file 11306_2020_1759_MOESM4_ESM.pdf]

**Metabolomics analysis reveals altered metabolites in lean compared with obese adolescents and additional metabolic shifts associated with hyperinsulinaemia and insulin resistance in obese adolescents: a cross-sectional study**

Elisabeth Müllner, Hanna E. Röhnisch, Claudia von Brömssen, Ali A. Moazzami*
*Corresponding author: Ali.Moazzami@slu.se; Department of Molecular Sciences, Swedish University of Agricultural Sciences, Uppsala, Sweden

**Online Resource 4:** *P*-values for effects of insulin, obesity glucose and their interactions with time after adjustment for gender and puberty

| Metabolite | Insulin | Obesity | Glucose | Time | Insulin*Time | Obesity*Time | Glucose*Time | Gender | Puberty |
|---|---|---|---|---|---|---|---|---|---|
| Valine | 0.0015[1] | 0.5502 | 0.2368 | <.0001 | 0.3631 | 0.0210 | 0.8300 | 0.1177 | 0.0195 |
| Leucine | 0.0002 | 0.7357 | 0.3805 | <.0001 | 0.2132 | 0.1275 | 0.9130 | 0.0130 | 0.0010 |
| Isoleucine | 0.0032 | 0.2215 | 0.2563 | <.0001 | 0.2909 | 0.6480 | 0.7374 | 0.0525 | 0.0316 |
| Tyrosine | 0.0028 | 0.1574 | 0.5104 | <.0001 | 0.1637 | 0.1830 | 0.9337 | 0.5167 | 0.4844 |
| Serine | 0.0456 | 0.8738 | 0.3579 | <.0001 | 0.5487 | 0.1200 | 0.8721 | 0.5176 | 0.2306 |
| Glycine | 0.4246 | 0.1772 | 0.7091 | <.0001 | 0.8921 | 0.2526 | 0.6305 | 0.1147 | 0.0898 |
| Myo-Inositol | 0.1235 | 0.9255 | 0.5642 | <.0001 | 0.7610 | 0.0071 | 0.4728 | 0.1787 | 0.5929 |
| Dimethylsulfone | 0.0256 | 0.3632 | 0.5219 | 0.9367 | 0.5324 | 0.0689 | 0.7551 | 0.0441 | 0.1071 |
| O-Acetylcarnitine | 0.2678 | 0.0005 | 0.8302 | <.0001 | 0.6085 | 0.0026 | 0.7970 | 0.1377 | 0.0403 |
| Glutamate | 0.8437 | 0.0343 | 0.3337 | 0.1887 | 0.0718 | 0.2267 | 0.1890 | 0.0969 | 0.7029 |
| Alanine | 0.1225 | 0.0577 | 0.6223 | 0.0374 | 0.8120 | 0.0315 | 0.8491 | 0.5056 | 0.3584 |
| Pyruvate | 0.7379 | 0.1299 | 0.2348 | <.0001 | 0.7892 | 0.0551 | 0.6792 | 0.7142 | 0.5830 |
| Acetate | 0.8484 | 0.0049 | 0.7905 | <.0001 | 0.7920 | 0.1381 | 0.4177 | 0.3841 | 0.1574 |
| Lysine | 0.0287 | 0.4657 | 0.6902 | <.0001 | 0.5439 | 0.1228 | 0.8984 | 0.2556 | 0.0007 |
| Phenylalanine | 0.0319 | 0.1719 | 0.0918 | <.0001 | 0.0814 | 0.2121 | 0.2762 | 0.9288 | 0.0003 |
| 2-Oxoisocaproate | 0.0283 | 0.5543 | 0.6091 | <.0001 | 0.3865 | 0.0951 | 0.4849 | 0.1138 | 0.0053 |

[1] linear mixed model (PROC MIXED) with time, insulin response (NI vs HI), obesity (lean vs obese), glucose responses (NGT vs IGT) and their interaction (time × insulin, time × obesity and time × glucose) as fixed factors and adjusted for gender and puberty.
