# Supplementary material for: Metabolomics analysis reveals altered metabolites in lean compared with obese adolescents and additional metabolic shifts associated with hyperinsulinaemia and insulin resistance in obese adolescents: a cross-sectional study
Source: Metabolomics. 2021 Jan 12;17(1):11. doi: 10.1007/s11306-020-01759-y (PMC7803706; doi:10.1007/s11306-020-01759-y)
Supplement: Supplementary file 5 — Supplementary material 5 (PDF 366.1 kb) [file 11306_2020_1759_MOESM5_ESM.pdf]

Metabolomics analysis reveals altered metabolites in lean compared with obese adolescents and additional metabolic shifts associated with hyperinsulinaemia and insulin resistance in obese adolescents: a cross-sectional study

Elisabeth Müllner, Hanna E. Röhnisch, Claudia von Brömssen, Ali A. Moazzami*
*Corresponding author: Ali.Moazzami@slu.se; Department of Molecular Sciences, Swedish University of Agricultural Sciences, Uppsala, Sweden

Online Resource 5: *P*-values for comparisons between the groups at each time point during the oral glucose tolerance test (0 – 120 min)

	0 min	5 min	10 min	15 min	30 min	60 min	90 min	120 min
**Valine**								
Lean, NI vs obese, NI	1.000	1.000	1.000	1.000	1.000	1.000	1.000	1.000
Lean, NI vs obese, HI	**0.003**	**0.003**	**0.001**	**0.005**	**0.001**	**0.000**	**0.001**	**0.000**
Lean, NI vs obese, HI + IGT	0.061	**0.013**	**0.005**	0.068	**0.007**	**0.004**	**0.005**	**0.001**
Obese, NI vs obese, HI	**0.042**	**0.012**	**0.002**	**0.010**	**0.013**	**0.009**	**0.006**	**0.011**
Obese, NI vs obese, HI + IGT	0.528	0.050	**0.015**	0.098	0.110	0.117	**0.021**	**0.036**
Obese, HI vs obese, HI + IGT	1.000	1.000	1.000	1.000	1.000	1.000	1.000	1.000
**Leucine**								
Lean, NI vs obese, NI	1.000	1.000	1.000	1.000	1.000	1.000	1.000	1.000
Lean, NI vs obese, HI	**0.001**	**0.003**	**0.001**	**0.002**	**0.000**	**0.002**	**0.049**	**0.034**
Lean, NI vs obese, HI + IGT	**0.020**	**0.018**	**0.004**	**0.040**	**0.005**	**0.009**	0.052	**0.027**
Obese, NI vs obese, HI	**0.008**	**0.003**	**0.000**	**0.007**	**0.002**	**0.013**	**0.009**	**0.012**
Obese, NI vs obese, HI + IGT	0.161	**0.017**	**0.002**	0.087	**0.043**	0.058	**0.010**	**0.009**
Obese, HI vs obese, HI + IGT	1.000	1.000	1.000	1.000	1.000	1.000	1.000	1.000
**Isoleucine**								
Lean, NI vs obese, NI	1.000	1.000	1.000	1.000	1.000	1.000	1.000	1.000
Lean, NI vs obese, HI	**0.002**	**0.002**	**0.000**	**0.002**	**0.000**	**0.001**	**0.015**	**0.016**
Lean, NI vs obese, HI + IGT	0.076	**0.006**	**0.004**	0.080	**0.009**	**0.008**	**0.023**	**0.008**
Obese, NI vs obese, HI	**0.037**	**0.021**	**0.001**	**0.022**	**0.022**	**0.029**	0.079	0.088
Obese, NI vs obese, HI + IGT	0.763	0.062	**0.010**	0.473	0.244	0.221	0.119	0.053
Obese, HI vs obese, HI + IGT	1.000	1.000	1.000	1.000	1.000	1.000	1.000	1.000
**Tyrosine**								
Lean, NI vs obese, NI	0.710	1.000	0.699	1.000	0.631	0.729	1.000	1.000
Lean, NI vs obese, HI	**0.000**	**0.000**	**0.000**	**<.0001**	**<.0001**	**<.0001**	**0.000**	**0.000**
Lean, NI vs obese, HI + IGT	**0.003**	**0.001**	**0.002**	**<.0001**	**0.001**	**0.000**	**0.001**	**0.002**
Obese, NI vs obese, HI	**0.043**	**0.028**	0.085	**0.001**	**0.017**	**0.029**	**0.009**	**0.032**
Obese, NI vs obese, HI + IGT	0.306	0.128	0.338	**0.002**	0.153	0.100	**0.018**	0.087
Obese, HI vs obese, HI + IGT	1.000	1.000	1.000	1.000	1.000	1.000	1.000	1.000

NI, normal insulin; HI, high insulin, IGT, impaired glucose tolerance;

Metabolomics analysis reveals altered metabolites in lean compared with obese adolescents and additional metabolic shifts associated with hyperinsulinaemia and insulin resistance in obese adolescents: a cross-sectional study

Elisabeth Müllner, Hanna E. Röhnisch, Claudia von Brömssen, Ali A. Moazzami*
*Corresponding author: Ali.Moazzami@slu.se; Department of Molecular Sciences, Swedish University of Agricultural Sciences, Uppsala, Sweden

Online Resource 5: *continued*

	**0 min**	**5 min**	**10 min**	**15 min**	**30 min**	**60 min**	**90 min**	**120 min**
**Serine**								
Lean, NI vs obese, NI	1.000	1.000	1.000	1.000	1.000	1.000	1.000	1.000
Lean, NI vs obese, HI	0.243	0.141	0.256	0.401	0.429	0.161	0.136	0.104
Lean, NI vs obese, HI + IGT	**0.009**	**0.020**	**0.005**	**0.032**	**0.035**	**0.023**	**0.035**	**0.007**
Obese, NI vs obese, HI	0.530	0.954	0.718	1.000	0.206	0.207	0.424	0.577
Obese, NI vs obese, HI + IGT	**0.029**	0.237	**0.031**	0.146	**0.015**	**0.034**	0.141	0.073
Obese, HI vs obese, HI + IGT	1.000	1.000	1.000	1.000	1.000	1.000	1.000	1.000
**Glycine**								
Lean, NI vs obese, NI	1.000	1.000	1.000	1.000	1.000	1.000	0.753	1.000
Lean, NI vs obese, HI	0.825	0.227	0.467	0.976	0.384	0.054	**0.042**	0.202
Lean, NI vs obese, HI + IGT	0.138	0.178	**0.045**	0.441	0.195	0.093	**0.010**	**0.042**
Obese, NI vs obese, HI	1.000	1.000	1.000	1.000	1.000	0.916	1.000	1.000
Obese, NI vs obese, HI + IGT	0.315	1.000	1.000	1.000	0.792	1.000	0.711	0.427
Obese, HI vs obese, HI + IGT	1.000	1.000	1.000	1.000	1.000	1.000	1.000	1.000
**Myo-Inositol**								
Lean, NI vs obese, NI	0.185	1.000	1.000	1.000	1.000	1.000	1.000	1.000
Lean, NI vs obese, HI	0.719	1.000	1.000	1.000	0.662	**0.012**	0.060	**0.030**
Lean, NI vs obese, HI + IGT	1.000	1.000	0.765	1.000	**0.029**	**0.001**	**0.012**	**0.003**
Obese, NI vs obese, HI	1.000	1.000	1.000	1.000	0.746	0.384	1.000	0.731
Obese, NI vs obese, HI + IGT	0.212	1.000	0.199	1.000	**0.041**	0.071	0.627	0.184
Obese, HI vs obese, HI + IGT	0.838	1.000	0.669	1.000	1.000	1.000	1.000	1.000
**Dimethylsulfone**								
Lean, NI vs obese, NI	1.000	0.382	1.000	0.676	1.000	1.000	1.000	1.000
Lean, NI vs obese, HI	0.185	**0.011**	0.249	1.000	0.483	**0.016**	**0.034**	**0.050**
Lean, NI vs obese, HI + IGT	0.112	**0.001**	**0.040**	1.000	0.076	0.060	**0.005**	**0.048**
Obese, NI vs obese, HI	0.876	1.000	1.000	0.080	0.744	**0.011**	0.818	0.476
Obese, NI vs obese, HI + IGT	0.620	0.405	0.501	0.508	0.145	**0.041**	0.240	0.486
Obese, HI vs obese, HI + IGT	1.000	1.000	1.000	1.000	1.000	1.000	1.000	1.000

NI, normal insulin; HI, high insulin, IGT, impaired glucose tolerance;

Metabolomics analysis reveals altered metabolites in lean compared with obese adolescents and additional metabolic shifts associated with hyperinsulinaemia and insulin resistance in obese adolescents: a cross-sectional study

Elisabeth Müllner, Hanna E. Röhnisch, Claudia von Brömssen, Ali A. Moazzami*
*Corresponding author: Ali.Moazzami@slu.se; Department of Molecular Sciences, Swedish University of Agricultural Sciences, Uppsala, Sweden

Online Resource 5: *continued*

	0 min	5 min	10 min	15 min	30 min	60 min	90 min	120 min
**O-Acetylcarnitine**								
Lean, NI vs obese, NI	**0.019**	0.109	0.044	**0.016**	**0.001**	**0.000**	0.062	**0.029**
Lean, NI vs obese, HI	0.061	0.493	0.166	0.086	**0.031**	0.073	0.302	0.520
Lean, NI vs obese, HI + IGT	0.394	1.000	0.148	0.068	**0.041**	0.172	0.466	0.712
Obese, NI vs obese, HI	1.000	1.000	1.000	1.000	1.000	0.477	1.000	1.000
Obese, NI vs obese, HI + IGT	1.000	1.000	1.000	1.000	1.000	0.184	1.000	0.853
Obese, HI vs obese, HI + IGT	1.000	1.000	1.000	1.000	1.000	1.000	1.000	1.000
**Glutamate**								
Lean, NI vs obese, NI	0.135	0.620	1.000	0.107	0.633	0.058	0.372	1.000
Lean, NI vs obese, HI	**0.019**	**0.008**	0.259	**0.047**	0.081	0.188	0.888	0.091
Lean, NI vs obese, HI + IGT	0.280	**0.003**	**0.017**	**0.025**	**0.030**	**0.011**	**0.048**	**0.005**
Obese, NI vs obese, HI	1.000	0.745	1.000	1.000	1.000	1.000	1.000	1.000
Obese, NI vs obese, HI + IGT	1.000	0.459	0.459	1.000	1.000	1.000	1.000	0.285
Obese, HI vs obese, HI + IGT	1.000	1.000	1.000	1.000	1.000	1.000	1.000	1.000
**Alanine**								
Lean, NI vs obese, NI	0.351	0.430	0.494	1.000	0.215	0.250	1.000	0.939
Lean, NI vs obese, HI	**0.005**	**0.003**	**0.007**	**0.013**	**0.004**	**0.005**	0.071	0.083
Lean, NI vs obese, HI + IGT	**0.011**	**0.008**	**0.045**	0.089	**0.029**	**0.023**	0.134	0.088
Obese, NI vs obese, HI	0.915	0.619	0.860	0.315	1.000	1.000	1.000	1.000
Obese, NI vs obese, HI + IGT	1.000	1.000	1.000	1.000	1.000	1.000	1.000	1.000
Obese, HI vs obese, HI + IGT	1.000	1.000	1.000	1.000	1.000	1.000	1.000	1.000
**Pyruvate**								
Lean, NI vs obese, NI	1.000	1.000	1.000	1.000	1.000	**0.041**	1.000	1.000
Lean, NI vs obese, HI	0.050	0.828	1.000	1.000	1.000	0.206	1.000	1.000
Lean, NI vs obese, HI + IGT	**0.036**	0.071	0.134	0.300	0.888	**0.024**	0.283	0.115
Obese, NI vs obese, HI	0.957	1.000	1.000	1.000	1.000	1.000	1.000	1.000
Obese, NI vs obese, HI + IGT	0.821	0.751	1.000	0.970	1.000	1.000	1.000	1.000
Obese, HI vs obese, HI + IGT	1.000	1.000	1.000	1.000	1.000	1.000	1.000	0.929

NI, normal insulin; HI, high insulin, IGT, impaired glucose tolerance;
