# Supplementary material for: Metabolomics analysis reveals altered metabolites in lean compared with obese adolescents and additional metabolic shifts associated with hyperinsulinaemia and insulin resistance in obese adolescents: a cross-sectional study
Source: Metabolomics. 2021 Jan 12;17(1):11. doi: 10.1007/s11306-020-01759-y (PMC7803706; doi:10.1007/s11306-020-01759-y)
Supplement: Supplementary file 6 — Supplementary material 6 (PDF 65.4 kb) [file 11306_2020_1759_MOESM6_ESM.pdf]

Metabolomics analysis reveals altered metabolites in lean compared with obese adolescents and additional metabolic shifts associated with hyperinsulinaemia and insulin resistance in obese adolescents: a cross-sectional study

Elisabeth Müllner, Hanna E. Röhnisch, Claudia von Brömssen, Ali A. Moazzami*
*Corresponding author: Ali.Moazzami@slu.se; Department of Molecular Sciences, Swedish University of Agricultural Sciences, Uppsala, Sweden

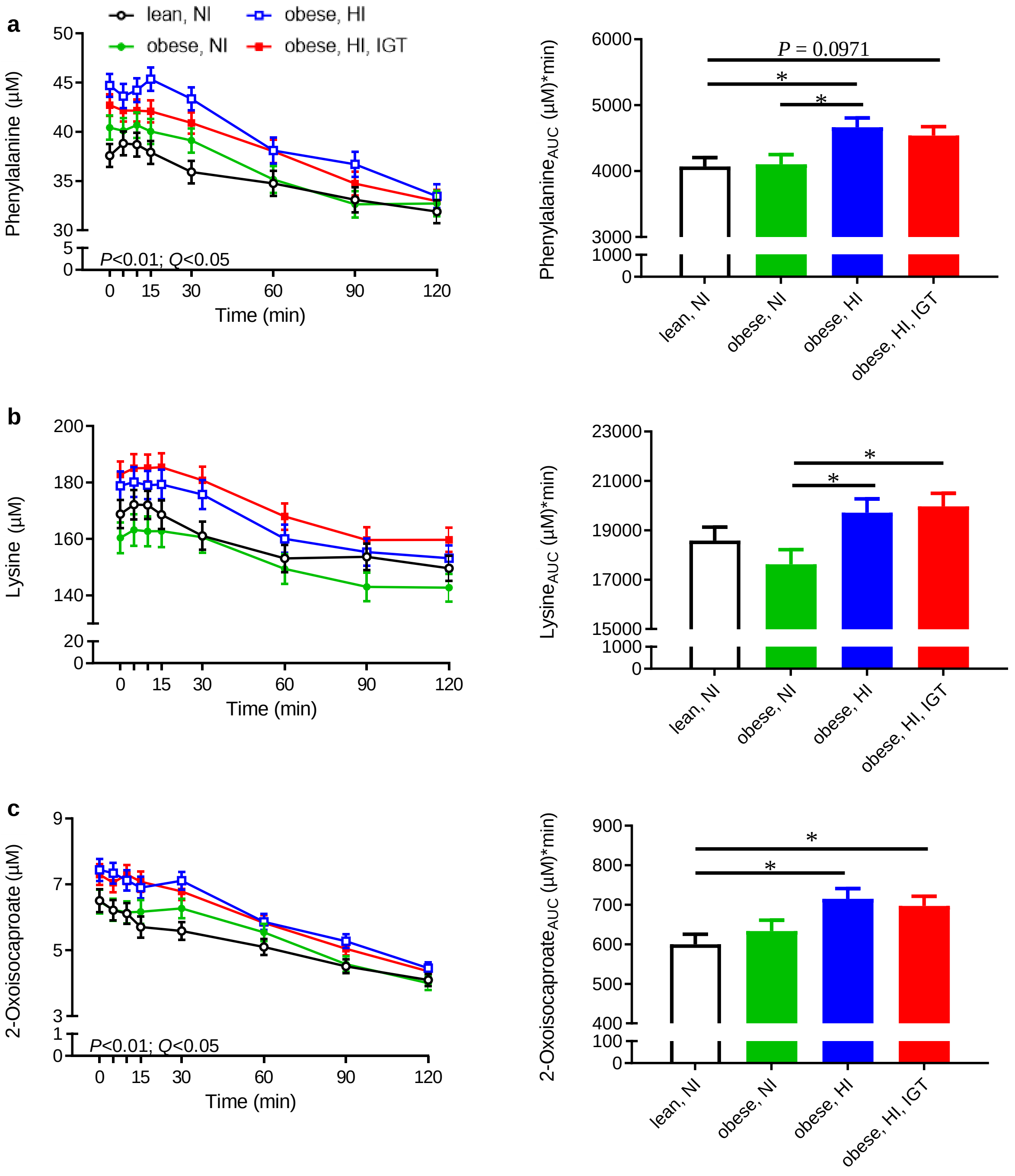

Online Resource 6: Plasma levels during an OGTT (left panel) and area under the curve (AUC; right panel) of (a) phenylalanine, (b) lysine and (c) 2-oxoisocaproate in lean adolescents with normal insulin (NI; black, n=21), adolescents with obesity and NI during OGTT (green, n=18), adolescents with obesity and high insulin (HI; blue, n=20), and adolescents with obesity and HI in combination with impaired glucose tolerance (IGT; red, n=23). *P*-values for group effect (n=4, mixed model) and corresponding Benjamini-Hochberg-adjusted *P*-values (*Q*-values) are presented. All metabolites also showed a significant insulin response effect (NI versus HI, $P<0.05$).
