# Supplementary material for: Metabolomics analysis reveals altered metabolites in lean compared with obese adolescents and additional metabolic shifts associated with hyperinsulinaemia and insulin resistance in obese adolescents: a cross-sectional study
Source: Metabolomics. 2021 Jan 12;17(1):11. doi: 10.1007/s11306-020-01759-y (PMC7803706; doi:10.1007/s11306-020-01759-y)
Supplement: Supplementary file 7 — Supplementary material 7 (PDF 159.6 kb) [file 11306_2020_1759_MOESM7_ESM.pdf]

Metabolomics analysis reveals altered metabolites in lean compared with obese adolescents and additional metabolic shifts associated with hyperinsulinaemia and insulin resistance in obese adolescents: a cross-sectional study

Elisabeth Müllner, Hanna E. Röhnisch, Claudia von Brömssen, Ali A. Moazzami*
*Corresponding author: Ali.Moazzami@slu.se; Department of Molecular Sciences, Swedish University of Agricultural Sciences, Uppsala, Sweden

Online Resource 7: Metabolic differences between groups after adjustment for gender and puberty.

Metabolite	HMDB-ID	Group[1]		Time	Group*Time[2]		Gender	Puberty
		P	***Q*[3]**		***P***	***Q*[3]**		
2-Aminobutyrate	0000452	0.7353	0.8007	<.0001	0.3431	0.4945	0.0948	0.0748
2-Hydroxybutyrate	0000008	0.3408	0.4912	<.0001	0.0154	0.0795	0.9586	0.8812
2-Hydroxyisovalerate	0000407	0.0239	0.0651	<.0001	0.0974	0.2651	0.0003	0.1563
2-Hydroxyvalerate	0001863	0.5855	0.6736	<.0001	**0.0017**	**0.0167**	0.6643	0.3933
2-Oxoisocaproate	0000695	**0.0112**	**0.0457**	<.0001	0.0098	0.0686	0.1145	0.0053
3-Hydroxybutyrate	0000011	0.0187	0.0611	<.0001	0.0204	0.0795	0.2185	0.7453
3-Methyl-2-oxovalerate	0000491	0.6947	0.7736	<.0001	0.9424	0.9424	0.1960	0.6267
Acetate	0000042	**0.0003**	**0.0025**	<.0001	0.0599	0.1954	0.3383	0.2683
Acetoacetate	0000060	0.0362	0.0887	<.0001	0.1280	0.3117	0.0015	0.9673
Acetone	0001659	0.4091	0.5162	<.0001	0.2135	0.3470	0.9792	0.0875
Alanine	0000161	**0.0047**	**0.0256**	0.0025	0.3287	0.4881	0.4903	0.2090
Arginine	0000517	0.4214	0.5162	<.0001	0.2009	0.3470	0.6649	0.1374
Asparagine	0000168	0.0152	0.0573	<.0001	0.0211	0.0795	0.2722	0.0022
Aspartate	0000191	0.9269	0.9462	0.0983	0.0638	0.1954	0.3761	0.4508
Betaine	0000043	0.9228	0.9462	<.0001	**<.0001**	**0.0002**	0.6753	0.1597
Carnitine	0000062	0.1218	0.2177	0.0006	0.2239	0.3470	0.0006	0.9895
Choline	0000097	0.4214	0.5162	<.0001	0.5721	0.6675	0.2868	0.8855
Citrate	0000094	0.1206	0.2177	<.0001	0.0203	0.0795	0.0206	0.0005
Creatine	0000064	0.9970	0.9970	<.0001	0.3842	0.5344	0.2185	0.4983
Creatinine	0000562	0.1282	0.2177	0.0004	0.6393	0.6810	0.5026	<.0001
Dimethylsulfone	0004983	**<.0001**	**0.0000**	0.1789	0.4253	0.5344	0.1340	0.0544
Ethanol	0000108	0.2242	0.3544	0.0002	0.4365	0.5347	0.2355	0.8526
Formate	0000142	0.0688	0.1466	0.0021	0.1403	0.3117	0.8537	0.5076
Glucose (NMR)	0000122	**0.0004**	**0.0028**	<.0001	**<.0001**	**0.0002**	0.3436	0.7929
Glutamate	0000148	**0.0090**	**0.0401**	0.0062	0.2266	0.3470	0.0868	0.8053

[1] Metabolites significantly different between the groups are highlighted in bold. Cut-off for Benjamini-Hochberg adjusted *P*-value is 0.0122.
[2] Metabolites with significant group*time interaction are highlighted in bold. Cut-off for Benjamini-Hochberg adjusted *P*-value is 0.0051
[3] Benjamini-Hochberg–adjusted *P* values are presented as *Q* values.

Metabolomics analysis reveals altered metabolites in lean compared with obese adolescents and additional metabolic shifts associated with hyperinsulinaemia and insulin resistance in obese adolescents: a cross-sectional study

Elisabeth Müllner, Hanna E. Röhnisch, Claudia von Brömssen, Ali A. Moazzami*
*Corresponding author: Ali.Moazzami@slu.se; Department of Molecular Sciences, Swedish University of Agricultural Sciences, Uppsala, Sweden

Online Resource 7: *continued*

Metabolite	HMDB-ID	Group[1]		Time	Group*Time[2]		Gender	Puberty
		P	***Q*[3]**		***P***	***Q*[3]**		
Glutamine	0000641	0.8550	0.9108	<.0001	0.6301	0.6810	0.2131	0.3673
Glycine	0000123	0.0907	0.1852	<.0001	0.6050	0.6810	0.2097	0.3790
Hippurate	0000714	0.4094	0.5162	0.3696	0.8816	0.9000	0.7865	0.0227
Histidine	0000177	0.3820	0.5162	<.0001	0.7447	0.7764	0.1661	0.0005
Isoleucine	0000172	**0.0002**	**0.0025**	<.0001	0.4631	0.5535	0.0762	0.0116
Isopropanole	0000863	0.2625	0.3898	0.4150	0.0145	0.0795	0.6520	0.3880
Lactate	0000190	0.2376	0.3638	<.0001	0.2021	0.3470	0.9179	0.0452
Leucine	0000687	**<.0001**	**0.0000**	<.0001	0.0936	0.2651	0.0238	0.0002
Lysine	0000182	0.1333	0.2177	<.0001	0.1479	0.3117	0.1549	0.0004
Methionine	0000696	0.0394	0.0919	<.0001	0.0181	0.0795	0.8636	0.0211
Myo-Inositol	0000211	0.0974	0.1909	<.0001	**<.0001**	**0.0002**	0.1989	0.2460
N,N-Dimethylglycine	0000092	0.5911	0.6736	<.0001	0.0074	0.0604	0.4212	0.7917
O-Acetylcarnitine	0000201	0.0166	0.0581	<.0001	0.0455	0.1593	0.0719	0.4018
Phenylalanine	0000159	**0.0065**	**0.0319**	<.0001	**0.0016**	**0.0167**	0.6338	0.0017
Proline	0000162	0.1330	0.2177	<.0001	0.1646	0.3117	0.0208	0.1352
Propionate	0000237	0.0236	0.0651	<.0001	0.4059	0.5344	0.8066	0.1859
Propylene glycol	0001881	0.0338	0.0872	<.0001	0.2165	0.3470	0.0051	0.2895
Pyruvate	0000243	0.0224	0.0651	<.0001	0.4148	0.5344	0.7692	0.4907
Sarcosine	0000271	0.3808	0.5162	0.0002	0.1395	0.3117	0.8920	0.5224
Serine	0000187	**0.0015**	**0.0092**	<.0001	0.6354	0.6810	0.6802	0.1327
Succinate	0000254	0.0537	0.1196	0.1561	0.4029	0.5344	0.3828	0.5191
Threonine	0000167	0.5040	0.6023	<.0001	0.1610	0.3117	0.0995	0.1089
Tyrosine	0000158	**<.0001**	**0.0000**	<.0001	0.1654	0.3117	0.6579	0.5030
Valine	0000883	**0.0003**	**0.0025**	<.0001	0.1611	0.3117	0.2245	0.0050

[1] Metabolites significantly different between the groups are highlighted in bold. Cut-off for Benjamini-Hochberg adjusted *P*-value is 0.0122.
[2] Metabolites with significant group*time interaction are highlighted in bold. Cut-off for Benjamini-Hochberg adjusted *P*-value is 0.0051
[3] Benjamini-Hochberg–adjusted *P* values are presented as *Q* values.
